# The effect of general anaesthesia on gastric myoelectric activity in experimental pigs

**DOI:** 10.1186/1471-230X-13-48

**Published:** 2013-03-14

**Authors:** Ilja Tachecí, Jaroslav Květina, Martin Kuneš, Michal Pavlík, Marcela Kopáčová, Vladimír Černý, Stanislav Rejchrt, Jithinraj Edakkanambeth Varayil, Jan Bureš

**Affiliations:** 1 2nd Department of Internal Medicine – Gastroenterology, Charles University in Prague, Faculty of Medicine and University Hospital, Hradec Králové, Czech Republic; 2 Department of Teaching Support, University of Defence, Faculty of Military Health Services, Hradec Králové, Czech Republic; 3 Department of Anaesthesiology, Resuscitation and Intensive Medicine, Charles University in Prague, Faculty of Medicine and University Hospital, Hradec Králové, Czech Republic; 4 Department of Anaesthesia, Dalhousie University, Halifax, Canada

**Keywords:** Electrogastrography, Experimental pigs, Gastric myoelectrical activity, General anaesthesia, Isoflurane, Nitrous oxide, Thiopental

## Abstract

**Background:**

Surface electrogastrography (EGG) is a non-invasive method for clinical assessment of gastric myoelectrical activity. Different forms of general anaesthesia might have various effects on porcine EGG. The aim of this study was to evaluate the impact of different anaesthetic agents on EGG in experimental pigs.

**Methods:**

Four 15-minute EGG intervals were recorded and analysed. A baseline EGG recording was started 20 minutes after intramuscular injection of ketamine and azaperone (periods A and B). Four different regimens of general anaesthesia followed immediately after the baseline EGG (5 pigs in each experimental group): thiopental, isoflurane, nitrous oxide and isoflurane plus nitrous oxide. EGG recordings followed for the next 30 minutes under general anaesthesia (periods C and D). The dominant frequencies of slow waves were compared between the baseline intervals A and B and periods C and D under general anaesthesia.

**Results:**

The mean dominant frequency was within the normal range (2.3 – 3.5 cycles per minute) in all animals in all regimens. Thiopental general anaesthesia did not influence any change of the dominant frequency of slow waves. Nitrous oxide general anaesthesia increased the dominant frequency of slow waves in a statistically significant manner (baseline: 2.93 ± 0.53 and 3.01 ± 0.53; under general anaesthesia: 3.25 ± 0.34 and 3.29 ± 0.38 cycles per minute; p < 0.001, p = 0.003, p < 0.001, p < 0.001). Nitrous oxide together with isoflurane induced a statistically significant decrease of dominant frequency in the last 15-minute interval (2.66 ± 0.55 cycles per minute) compared to the baseline recording (2.81 ± 0.49; p = 0.030).

**Conclusions:**

All changes of porcine gastric myoelectric activity assessed by the dominant frequency of slow waves during EGG remained within the normal range although some of them achieved statistical significance. Thus all tested agents used for general anaesthesia can be recommended in preclinical studies with porcine models focused on gastric myoelectric activity without any risk of compromising the results. Thiopental seems to be the most suitable as it did not cause any changes at all.

## Background

Surface electrogastrography (EGG) is a non-invasive method for clinical assessment of gastric myoelectrical activity [1,2]. Our group has demonstrated that EGG is also reliable and feasible in experimental pigs [3,4]. Porcine EGG is fully comparable with that recorded in healthy humans [2,3,5].

Pigs can be used in various preclinical experiments [5-9] as an omnivorous representative due to their relatively very similar gastrointestinal functions compared to humans [10]. However, all these studies must be performed under general anaesthesia in experimental pigs. Limited data were published so far, showing that general [11,12] and combined spinal-epidural anaesthesia [13] may affect myoelectrical activity of the stomach in humans. However, the exact mechanism of surgery and anaesthesia on gastric myoelectric activity is less clear [14].

Several experiments with invasive monitoring of porcine gastrointestinal motility have been published to date [15,16]. To the best of our knowledge, there are no reports dealing with the effect of different types of general anaesthesia on porcine EGG in the available literature. Our hypothesis stated that different anaesthesia might have various effects on porcine gastric myoelectrical activity. The aim of this study was to evaluate the impact of different anaesthetic agents on EGG in experimental pigs and to compare the results with published human data.

## Methods

### Animals

Mature young female experimental pigs (*Sus scrofa f. domestica*, hybrids of Czech White and Landrace breeds, median weight 31.0 kg) entered the study. The animals were fed twice a day (standard assorted food A1) and were allowed unrestricted access to water. All the pigs were randomly assigned to particular study groups.

The Project was approved by the Institutional Review Board of the Animal Care Committee of the University of Defence, Faculty of Military Health Services, Hradec Králové, Czech Republic (Protocol Number 35-14/2012-3696). The animals were held and treated in accordance with the European Convention for the Protection of Vertebrate Animals Used for Experimental and Other Scientific Purposes [17].

### Experimental design

All EGG recordings were accomplished in the morning after 24 hours of fasting. Intramuscular injections of ketamine (20 mg per kg; Narkamon, Spofa, Prague, Czech Republic) and azaperone (2.2 mg per kg; Stresnil, Janssen Animal Health, Saunderton, UK) were used as an introduction to anaesthesia in all animals. A baseline EGG recording started 20 minutes after this intramuscular injection (two 15-minute intervals: periods A and B).

Four different regimens of general anaesthesia followed immediately after the baseline EGG recording (5 animals in each experimental group):

**Group 1:** Thiopental (Thiopental Valeant, Valeant Czech Pharma s.r.o., Prague, Czech Republic); i.v. infusion 200 mg hour ^-1^, in five pigs (weighing 30.9 ± 1.6; median 31.5 kg);

**Group 2:** Isoflurane (Flurane, Abbott, Queenborough, UK) delivered by mask: inhalation 2% isoflurane in medicinal oxygen (2 litres per minute), in five pigs (weighing 30.2 ± 2.5; median 31.0 kg);

**Group 3:** Nitrous oxide (Linde Gas a.s., Prague, Czech Republic) delivered by mask: nitrous oxide (2 litres per minute) in medicinal oxygen (1 litre per minute), in five pigs (weighing 30.2 ± 2.4; median 30.0 kg);

**Group 4:** Isoflurane plus nitrous oxide in medicinal oxygen (in ratio 0.6 : 1 : 1) delivered by mask: 2 litres per minute, in five pigs (weighing 31.5 ± 1.3; median 31.0 kg).

Because of potential influence of artificial ventilation on the gastric myoelectric activity, all investigations were performed without endotracheal intubation in spontaneously breathing animals. EGG recordings followed for the next 30 minutes under general anaesthesia (periods C and D).

### Electrogastrography

**Figure 1:**
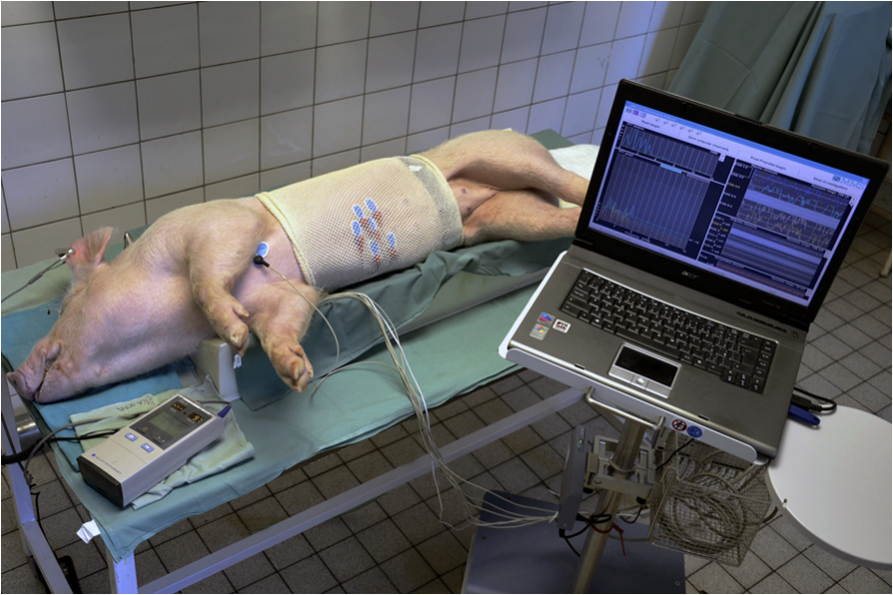
General view of the arrangement of electrogastrography in an experimental pig.

**Figure 2:**
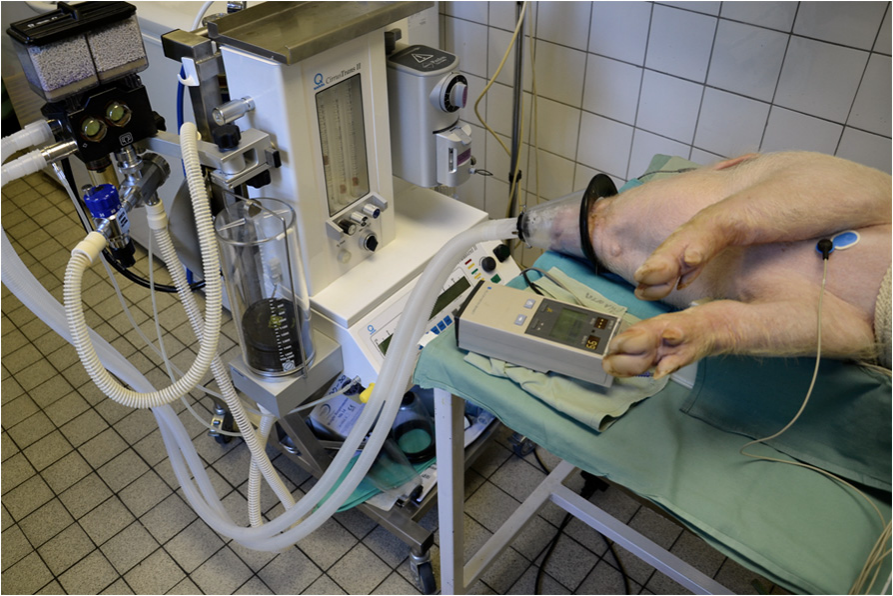
View of general inhalation anaesthesia delivered by mask.

**Figure 3:**
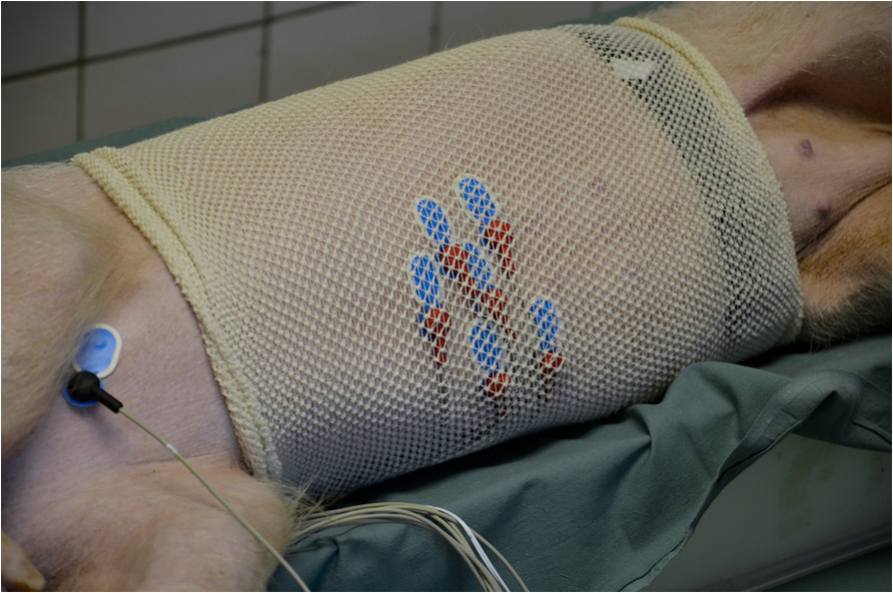
Detailed view of placement of electrodes and special abdominal belt (functioning as a respiratory sensor).

All animals were lying in a right lateral position during EGG recording. The epigastric area was shaved first and the skin was gently sandpapered afterwards. Six active self-adhesive electrodes were placed on the upper part of the abdomen, the 7th electrode (basal) was placed left of the mid-sternum. A special abdominal belt (respiratory sensor) was used to identify possible artefacts due to breathing and body movements (see Figures 1, 2 and 3 for general arrangement).

Surface cutaneous EGG was recorded using an Electrogastrography Stand Alone System (MMS – Medical Measurement Systems B.V., Enschede, the Netherlands).

MMS software (version 8.19) was used to assess EGG recordings. Running spectral analysis (based on Fourier transform) was used for elemental evaluation of the EGG. The results were expressed as running spectrum percent activity and the dominant frequency of slow waves was set at all intervals of EGG recordings.

### Statistical analysis

Data were statistically treated by means of descriptive statistics and Kruskal-Wallis One Way Analysis of Variance (ANOVA) using SigmaStat software (version 3.1, Jandel Corp., Erkrath, Germany). The dominant frequency of slow waves was displayed as median and interquartile range in the Table 1. To evaluate changes of the dominant frequency of slow waves, comparison of baseline periods (A and B) and periods under general anaesthesia (C and D) was performed.

**Table 1:** Electrogastrography in young mature female experimental pigs

**Parameter**	**Period A**	**Period B**	**Period C**	**Period D**	**Statistical significance** *
**Thiopental**	2.58	2.81	2.34	2.58	NS
n = 5	2.11 – 3.52	2.11 – 3.28	2.11 – 3.28	2.11 – 3.28	
**Isoflurane**	3.05	3.05	3.28	3.28	NS
					NS
n = 5	2.81 – 3.75	2.58 – 3.52	3.05 – 3.75	2.58 – 3.52	p = 0.004
					NS
**Nitrous oxide**	3.05	3.05	3.28	3.28	p < 0.001
					p < 0.001
n = 5	2.58 – 3.28	2.70 – 3.28	2.81 – 3.52	3.05 – 3.52	p = 0.003
					p < 0.001
**Nitrous oxide + isoflurane**	2.58	2.81	2.70	2.58	NS
					p = 0.030
n = 5	2.58 – 3.28	2.34 – 3.28	2.34 – 3.28	2.11 – 3.05	NS
					p = 0.037

Period A: a 15-minute baseline EGG recording was started 30 minutes after intramuscular injection of ketamine and azaperon.

Period B: a 15-minute baseline EGG recording was started 45 minutes after intramuscular injection of ketamine and azaperon.

Period C: a 15-minute EGG recording under continuous general anaesthesia (started immediately after the beginning of general anaesthesia).

Period D: a 15-minute EGG recording under continuous general anaesthesia (started 15 minutes after the beginning of general anaesthesia).

* Statistical significance: Period A vs. C, A vs. D, B vs. C, B vs. D (using Kruskal-Wallis One Way Analysis of Variance).

NS - not significant.

Dominant frequency of slow waves (cycles per minute), expressed as median and inter-quartile range.

## Results

EGG recording was successfully accomplished in all regimens in all animals. Based on our current resting EGG recordings in fasting animals, the normal dominant frequency of slow waves in young mature female experimental pigs is 2.9 ± 0.6 cycles per minute (confidence interval of the mean 0.05, median 2.8, inter-quartile range: 2.3 – 3.3). There was remarkable inter-individual variability within this normal range among animals in particular study groups (see Table 1 for details). The normal resting heart rate is 73.7 ± 9.7 beats per minute. There was no significant relationship between the basal dominant frequency of gastric slow waves and either the resting heart rate or body weight of the animals.

Thiopental general anaesthesia did not influence any change of the dominant frequency of slow waves. Nitrous oxide general anaesthesia increased the dominant frequency of slow waves in a statistically significant manner (baseline: 2.93 ± 0.53 and 3.01 ± 0.53; under general anaesthesia: 3.25 ± 0.34 and 3.29 ± 0.38 cycles per minute; p < 0.001, p = 0.003, p < 0.001, p < 0.001). Nitrous oxide together with isoflurane induced a statistically significant decrease of dominant frequency in the last 15-minute interval (2.66 ± 0.55 cycles per minute) compared to the baseline recording (2.81 ± 0.49; p = 0.030) (see Table 1 for details).

The heart rate was significantly higher under thiopental general anaesthesia compared to resting values (70.2 ± 11.5 vs. 89.7 ± 20.3; p < 0.001). Isoflurane anaesthesia produced a non-significant trend toward higher heart rate compared to resting values (81.4 ± 7.7 vs. 82.5 ± 8.3; p = 0.077). There was weak but statistically significant negative correlation between the dominant frequency of gastric slow waves and the heart rate under isoflurane general anaesthesia (r = − 0.451; p < 0.001).

## Discussion

To the best of our knowledge, this is the first study focused on the impact of different anaesthetics on EGG in experimental pigs.

As no studies concerning the effect of general anaesthesia on porcine EGG are available, we can compare our results with those limited data obtained in humans. To date, only a few studies have dealt with the impact of general anaesthesia on myoelectric activity of the stomach in humans, with variable results [12,13]. Cheng et al. [11] evaluated combined general anaesthesia in children (undergoing non-abdominal surgery), using thiopental (5 mg kg^-1^), isoflurane (1.5%), nitrous oxide (70%) and oxygen (30%). Tachygastria (associated with nausea and vomiting) became prominent immediately after induction and returned to normal 90 minutes after cessation of general anaesthesia. Lombardo et al. [12] studied sevoflurane (together with remifentanyl and cisatracurium besylate) in elective aortic surgery. There was no change in the dominant frequency during EGG, but the power ratio after surgery was significantly higher in patients with combined general plus epidural anaesthesia compared to the group of general anaesthesia alone.

Inhaled anaesthetics modify electrical activity of the central nervous system as measured by electroencephalography (EEG) [18], however there is little knowledge about the possible impact on myoelectrical activity of the stomach. In humans, nitrous oxide general anaesthesia is associated with a risk of postoperative nausea and vomiting, apart from other risk factors, interestingly also with motion sickness in the patient’s history [19,20]. The pathogenesis of postoperative nausea and vomiting is still largely unclear. Several drugs, recommended for prevention of postoperative nausea and vomiting (due to nitrous oxide anaesthesia) could have an impact on myoelectric activity of the stomach, influencing serotonin 5-HT3 receptors (setrons), dopamine D2 receptors (droperidol, metoclopramide), muscarinergic acetylcholine receptors (scopolamine) or neurokinin NK1 receptors (aprepitant) [19]. Total intravenous anaesthesia is associated with less postoperative nausea and vomiting compared to inhalational anaesthesia [21]. The analgesic effect of nitrous oxide is mediated by endogenous mediators, including opioids in nature [22]. It is well known that morphine influences gastric emptying and myoelectric activity, making regular slow wave rhythm in the antrum significantly lower in humans [23,24]. Similarly, fentanyl decreased dominant frequency of the gastric slow waves but only in about half of the human patients [25].

Our results indicated slight but significant increase in dominant frequency of gastric slow waves in general anaesthesia based on nitrous oxide. Nevertheless we are fully aware that all these data must be interpreted with caution. All changes identified in dominant frequency were only minor and mostly fluctuated within the normal range. None of the results reached the zone of tachygastria in comparison with the results of human studies [11].

Furthermore, there was considerable variability among individual pigs using particular study regimens. We did not observe any symptoms of gastric motility changes (vomiting) in any animal after general anaesthesia.

Drug interactions may reveal mechanisms of drug action: additive interactions suggest a common site of action, and synergistic interactions suggest different sites of action. Between drug classes, most interactions were synergistic. The major exception is ketamine, which typically interacts in either an additive or infra-additive (antagonistic) manner. Ketamine may increase NO release independent of acetylcholin regulation, therefore gastric motility might be theoretically and indirectly affected via nitric oxide pathway. DIn our current study, ketamine might not influence the overall results as it was administered in the same dose and manner in all regimens of subsequent general anaesthesia. Dopamine-antagonists like azaperone might have a potential effect to stabilise control of gastric motility as dopamin-antagonists have an antiemetic effect. Thereby azaperone might help to maintain a normal electrogastrograhic pattern and thus influence all of the results. Searching for an optimal anaesthetic regimen for preclinical EGG studies in experimental pigs, this possible effect would be greatly beneficial. Unlike phenothiazines, azaperone (a derivative of butyrophenone) has minimal anticholinergic effects. Similarly to ketamine, azaperone was administered in the same dose and manner in all regimens of subsequent general anaesthesia in our current study. This is why it might not influence the overall results significantly.

Nitrous oxide together with isoflurane induced a statistically significant decrease of dominant frequency in the last 15-minute interval compared to the baseline recording in our current study. The explanation of this phenomenon is not clear. Inhaled anaesthetics typically show synergy with i.v. anaesthetics, but are additive or, in the case of nitrous oxide and isoflurane, possibly infra-additive with each other [26]. Our current study is important as it produced new original data. All preclinical studies in experimental pigs must be carried out under general anaesthesia. The possible effect of anaesthetics and drug interaction must always be considered.

## Conclusion

In conclusion: All changes of porcine gastric myoelectric activity assessed by the dominant frequency of slow waves during EGG remained within the normal range although some of them achieved statistical significance. Thus all tested agents used for general anaesthesia can be recommended in preclinical studies with porcine models focused on gastric myoelectric activity without any risk of compromising the results. Thiopental seems to be the most suitable as it did not cause any changes at all.

## Abbreviations

EGG: Electrogastrography.

## Competing interests

The authors declare that they have no competing interest.

## Authors’ contributions

IT: created the study design, carried out the experimental part of the study – EGG, prepared analysis and interpretation of data, reviewed the literature and compiled the manuscript. JK: carried out the experimental part of the study – EGG and participated in the design of the study. MK: carried out the experimental part of the study – anaesthesiology. MP: carried out the experimental part of the study – anaesthesiology. MK: carried out the experimental part of the study, analysis and interpretation of data. VC: participated in study design and helped to draft the manuscript. SR: helped with analysis and interpretation of data, JEV: helped with EGG evaluation and statistical analysis. JB: conceived the study, participated in its design and coordination, carried out the experimental part of the study, performed the statistical analysis, contributed to analysis and interpretation of data and helped to draft the manuscript. All of the authors read and approved the final manuscript.

## Pre-publication history

The pre-publication history for this paper can be accessed here:

http://www.biomedcentral.com/1471-230X/13/48/prepub
